# Multi-Parameter Measurement of Rotors Using the Doppler Effect of Frequency-Swept Interferometry

**DOI:** 10.3390/s20247178

**Published:** 2020-12-15

**Authors:** Bin Shao, Wei Zhang, Peng Zhang, Weimin Chen

**Affiliations:** 1 Key Laboratory of Optoelectronic Technology and Systems, Ministry of Education, Chongqing University, Chongqing 400044, China; bshao@cqu.edu.cn (B.S.); zhangpeng@cqu.edu.cn (P.Z.); wmchen@cqu.edu.cn (W.C.); 2 Artificial Intelligence Center, Peng Cheng Laboratory, Shenzhen 518000, China

**Keywords:** rotor, multi-parameter, frequency-swept interferometry, Doppler Effect

## Abstract

The Doppler effect of frequency-swept interferometry (FSI) is often seen as an obstacle to the dynamic ranging accuracy. However, the potential of this obstacle is rarely noticed and used. In this paper, by combining the periodical characteristics of the rotational Doppler effect, an FSI-based multi-parameter measurement method for the rotor is proposed. Through the establishment of the rotational Doppler formula of FSI, it is found that the frequency, direct component, and amplitude of the dynamic distance given by FSI can be utilized to estimate the angular velocity, axial clearance, and tilt angle of the rotor, respectively. A rotor platform and a fiber-optic FSI system were constructed, and a series of experiments were carried out to verify the proposed method. The experimental results showed that the relative errors of the measured axial clearance, angular velocity, and tilt angle were less than 3.5%. This work provides a new perspective on the multi-parameter measurement of the rotor and makes it possible to directly perform multi-parameter measurement inside the space-confined rotating machinery as only a single small-size fiber-optic probe is needed.

## 1. Introduction

Rotors are the core component of various rotating machines, such as motors, generators, air compressors, wind turbines, and aero engines. Although these machines are designed for different purposes, their running efficiency is mainly determined by the rotor parameters [1,2]. Therefore, the measurement of rotor parameters is of great importance for performance evaluation [3,4], fault diagnostics [5,6], health monitoring [7,8,9,10], fatigue life prediction [11,12], and dynamic control [13,14] of rotating machines.

**Figure 1:**
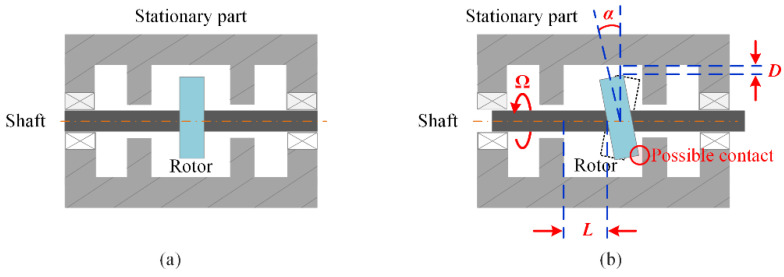
(**a**) The ideal case, and (**b**) the real situation of the running rotor. (*L*: axial clearance; *D*: radial clearance; *α*: tilt angle; Ω: angular velocity.).

The ideal running state of the rotor is shown in Figure 1a, where there is no contact-rubbing between the rotor and the stationary part. However, the real situation, as shown in Figure 1b, is that the rotor will deviate from the optimal operating state due to the manufacturing error, assembly tolerance, imbalance, and other complex mechanisms. Such deviation may result in different damages ranging from cosmetic damage to full destruction of the machine. To know and master the actual working state of the rotor, the synchronous measurement of the rotational speed Ω, tilt angle of the rotor *α*, and clearances *L* and *D* between the rotor and stationary part is necessary. To realize the measurement of these parameters, the conventional way is to integrate a number of individual subsystems, and each subsystem is dedicated to measuring a single particular parameter. Because each subsystem has its own sensor and corresponding demodulation equipment, the integrated system has a high cost and low overall reliability. Furthermore, the multi-sensor configuration requires sufficient space to allow the access and installation of multiple sensors, in practice, which is a large difficulty for space-confined rotating machines. Besides, as a side effect, the load or stress imposed by multiple sensors may, in turn, affect the running status of machines. Thus, to overcome the above challenges and better serve various applications, it is highly desired to develop a multi-parameter measurement method with fewer sensors. At present, a wide range of techniques have been reported on for the measurement of radial clearance *D* [15,16,17,18,19,20,21,22]. Among these techniques, the fiber-optic-based sensing approaches [17,18,19,20,21,22] are especially favored because of the intrinsic capabilities of optic fiber, such as its light weight, small size, and adaptability for embedding into structures. However, due to the narrow internal space and harsh measurement environment of the rotating machines, the measurement of the tilt angle and axial clearance under high-speed rotation is still a difficult problem that remains unsolved.

As a famous absolute distance measurement technique, frequency-swept interferometry (FSI) is widely used in various ranging applications. Since the dynamic accuracy of FSI is severely restricted by the well-known Doppler effect [23,24,25], the previously reported works mainly focused on the elimination of the Doppler error of FSI [23,24,25,26,27,28,29,30,31,32]. Because the goal of these methods was to measure the absolute dynamic distance, they were not applied for the measurement of the rotor parameters. In our previous work [32], we proposed an FSI-based method which is suitable for dynamic clearance measurement, but the core idea is still to remove the Doppler error in FSI. For absolute distance measurement, the Doppler effect is an undesirable property that needs to be eliminated, but we found, it is exactly this property that makes the multi-parameter measurement of the rotor possible. If the probe of the FSI is pointed to the rotor disk, the parameters *L*, Ω, and *α* become three factors that modulate the frequency, direct component, and amplitude of the dynamic distance given by FSI. Thus, by reversely using the characteristics of the dynamic distance, the rotor parameters *L*, Ω, and *α* can be simultaneously obtained according to the modulation relationship. Different from the thought of Doppler effect elimination, here we take the Doppler effect as a tool and utilize it to address the unresolved issues in rotor measurement. To realize the multi-parameter measurement, we first derived the modulation relationship between the Doppler effect and the parameters *L*, Ω, and *α*. Based on the modulation relationship, the estimation method of *L*, Ω, and *α* was given. In addition, experiments were conducted to prove the effectiveness of the estimation method. By using this method, the *L*, Ω, and *α* of rotors could be determined with a simple and low-cost FSI system, and as this system requires a single fiber-optic sensing probe, it has a great advantage for the multi-parameter measurement inside the space-confined rotating machines.

The remaining sections of this paper are organized as follows: In Section 2.1, the problem of the Doppler effect of FSI is reviewed. In Section 2.2, we model the Doppler effect of the rotor and propose a method to estimate the rotor parameters *L*, Ω, and *α*. Also, we analyze the impacts of the deflection angles caused by the non-parallel installation of the probe on the multi-parameter measurement error and give guidance for the probe installation location. Section 3.1 describes the experimental setup, and Section 3.2 presents the experimental procedure to validate the multi-parameter measurement method. Finally, concluding remarks are given in Section 4. In addition, Appendix A demonstrates the derivation process of the upper bound of the relative error in the estimation of *α*.

## 2. Theory

### 2.1. The Basics of FSI

**Figure 2:**
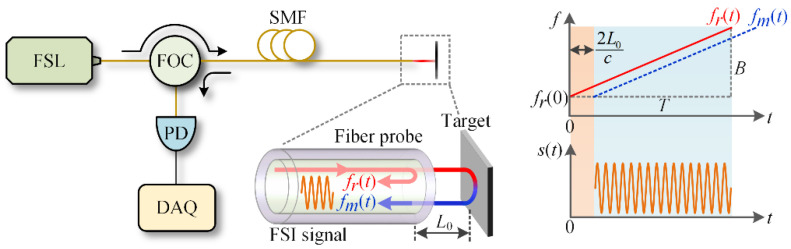
Schematic of the fiber-optic FSI system. (FSL: frequency-swept laser; FOC: fiber-optic circulator; SMF: single-mode fiber; PD: photodetector; DAQ: data acquisition system.).

A fiber-optic FSI system is shown in Figure 2; the light wave of the frequency-swept laser (FSL) passes through the fiber-optic circulator (FOC) and reaches the end face of the fiber probe. After the reflections of fiber probe and target, the back-propagating waves (i.e., the reference wave with frequency *f_r_*(*t*), and the measurement wave with frequency *f_m_*(*t*)) interfere and generate the FSI signal in the single-mode fiber (SMF), as shown in the enlarged figure. Then the FSI signal is routed to the photodetector (PD) by the FOC and finally sampled by the data acquisition system (DAQ). For a static target at distance *L*_0_, the FSI signal *s*(*t*) can be expressed as [30]

(1) $$\begin{array}{cc} s\left(t\right) & =\cos\left\{2\pi \int_{0}^{t}\left[f_{r}\left(t\right)-f_{m}\left(t\right)\right]dt\right\}\\ & =\cos\left\{2\pi \int_{0}^{t}\left[f_{r}\left(0\right)+\frac{B}{T}t-f_{r}\left(0\right)-\frac{B}{T}\left(t-\frac{2L_{0}}{c}\right)\right]dt\right\}\\ & =\cos\left[2\pi \left(\frac{B}{T}\frac{2L_{0}}{c}\right)t\right]=\cos\left[\psi \left(t\right)\right], \end{array}$$

where *f_r_*(*t*) = *f_r_*(0) + (*B*/*T*)*t*, *f_m_*(*t*) = *f_r_*(0) + (*B*/*T*)(*t* − 2*L*_0_/*c*), *B* is the sweep bandwidth of FSL, *T* is the sweep period of FSL, and *c* is the speed of light in air. Thus, using the phase *ψ*(*t*) of the measured *s*(*t*), the *L*_0_ can be determined as (*dψ*(*t*)/*dt*)·*Tc*/(4*πB*).

However, if the target moves during the frequency sweep, due to the Doppler effect, the phase *ψ*(*t*) in Equation (1) becomes [24] (note that, Jia et al. [24] gives the instantaneous frequency of the FSI signal, which equals *dψ*(*t*)/(2*πdt*))

(2) $$\psi \left(t\right)=\frac{4\pi }{c}\int_{o}^{t}\left[\frac{B}{T}L\left(t\right)+f_{r}\left(t\right)v\left(t\right)\right]dt,$$

where *L*(*t*) and *v*(*t*) are the real-time distance and velocity (*v*(*t*) is negative when the target approaches the fiber probe). Thus, using the *dψ*(*t*)/*dt*, the measured dynamic distance *L_m_*(*t*) can be obtained as [32]

(3) $$L_{m}\left(t\right)=\frac{Tc}{2B}\frac{d\psi \left(t\right)}{2\pi dt}=L\left(t\right)+\frac{f_{r}(t)}{B}v\left(t\right)T\approx L\left(t\right)+\frac{f_{avg}}{B}v\left(t\right)T,$$

where *f_avg_* is the average of *f_r_*(*t*). In Equation (3), the term *f_avg_v*(*t*)*T*/*B* is the Doppler error, which is sensitive to the target velocity *v*(*t*). This error severely restricts the dynamic ranging accuracy and needs to be eliminated. Whereas from the opposite perspective, the *L_m_*(*t*) may be utilized since it contains both the information of *L*(*t*) and *v*(*t*).

### 2.2. Multi-Parameter Measurement Method for the Rotor

**Figure 3:**
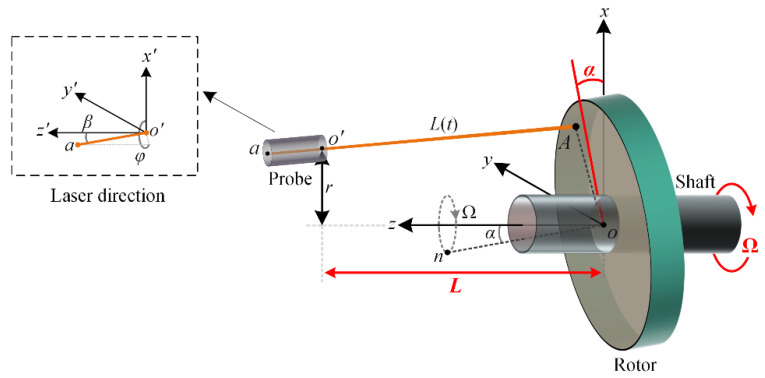
Schematic of the multi-parameter measurement of the rotor.

The general case of the multi-parameter measurement is shown in Figure 3. We assumed that the center of the rotor locates at the origin *o* (0, 0, 0) of the Cartesian coordinate system *xyz*, and that the rotor rotates around the *z*-axis with angular velocity Ω. Due to the tilt angle *α*, the normal vector of the rotor $\vec{\mathrm{on}}=[–\sin\alpha \cos(\Omega t),\;–\sin\alpha \sin(\Omega t),\;\cos\alpha ]$ will rotate around the *z*-axis with angular velocity Ω and angle *α*. We assumed the fiber probe is installed at an off-axis position *o*’ (*r*, 0, *L*), and the direction of the laser beam is $\vec{o^{\prime }a\;}=\;[\sin\beta \cos\varphi ,\;\sin\beta \sin\varphi ,\;\cos\beta ]$, where *β* and *φ* are the deflection angles between the laser beam and the coordinate system *x*’*y*’*z’* (parallel to *xyz*). Let the coordinates of point *A* be (*x_A_*, *y_A_*, *z_A_*), then we have $\vec{\mathrm{oA}}\;=\;[x_{A},\;y_{A},\;z_{A}]$ and $\vec{o\prime A}\;=\;[x_{A}-r,\;y_{A},\;z_{A}-L]$. Thus, according to the relations of $\vec{\mathrm{on}}⊥\vec{\mathrm{oA}}$ ($\vec{\mathrm{on}}\cdot \vec{\mathrm{oA}}\;=\;0$) and $\vec{o\prime a}||\vec{o\prime A}$ ($\sin\beta \cos\varphi /(x_{A}\;–\;r)\;=\;\sin\beta \sin\varphi /y_{A}\;=\;\cos\beta /(z_{A}-L)$), the following equations involving the angular velocity Ω, axial clearance *L*, and tilt angle α can be established.

(4) $$\left[\begin{array}{ccc} -\sin\alpha \cos(\Omega t) & -\sin\alpha \sin(\Omega t) & \cos\alpha \\ 1 & 0 & -\tan\beta \cos\varphi \\ 0 & 1 & -\tan\beta \sin\varphi \end{array}\right]\left[\begin{array}{l} x_{A}\\ y_{A}\\ z_{A} \end{array}\right]=\left[\begin{array}{c} 0\\ r-L\tan\beta \cos\varphi \\ -L\tan\beta \sin\varphi \end{array}\right].$$

Note that, the first line of Equation (4) is the result of $\vec{\mathrm{on}}\cdot \vec{\mathrm{oA}}\;=\;0$, the second and third lines are the equivalent transformations of $\sin\beta \cos\varphi /(x_{A}\;–\;r)\;=\;\sin\beta \sin\varphi /y_{A}\;=\;\cos\beta /(z_{A}\;–\;L$).

According to Equation (4), *z_A_* can be calculated as:

(5) $$z_{A}=\frac{r\sin\alpha \cos(\Omega t)-L\sin\alpha \tan\beta \cos(\Omega t-\varphi )}{\cos\alpha -\sin\alpha \tan\beta \cos(\Omega t-\varphi )}.$$

Using the angle *β* between the $\vec{o\prime a}$ and the $\vec{o\prime z\prime }$, the real dynamic clearance *L*(*t*) can be calculated as:

(6) $$L\left(t\right)=\left|o\prime A\right|=\sec\beta \left(L-z_{A}\right).$$

Then, substituting *L*(*t*) and *v*(*t*) = *∂L*(*t*)/∂*t* into Equation (3), the dynamic clearance given by the FSI can be expressed as

(7) $$\begin{array}{cc} L_{Ro}\left(t\right) & =L(t)+\frac{f_{avg}T}{B}\frac{\partial L(t)}{\partial t}=L(t)-\frac{f_{avg}T}{B}\frac{\partial \left(z_{A}\sec\beta \right)}{\partial t}\\ & =\frac{L+r\tan\alpha \sqrt{1+{\left(\frac{f_{avg}T\Omega }{B}\right)}^{2}}sin\left(\Omega t+\xi \right)-\frac{f_{avg}T\Omega }{B}\left(L-z_{A}\right)\tan\alpha \tan\beta \sin(\Omega t-\varphi )}{\cos\beta \left[1-\tan\alpha \tan\beta \sin(\Omega t-\varphi )\right]}, \end{array}$$

where subscript *Ro* denotes rotor, and:

(8) $$\xi =\arctan\left(\frac{-B}{f_{avg}T\Omega }\right).$$

From Equation (7), it can be found that the probe installation parameters *r, β* and *φ*, and the rotor parameters *L*, *α,* and Ω, are involved in *L_Ro_*(*t*). For a parallel laser beam, i.e., *β* ≈ 0, Equation (7) can be reduced as:

(9) $$L_{Ro}\left(t\right)=L+\underset{A_{Ro}}{\underset{︸}{r\tan\alpha \sqrt{1+{\left(\frac{f_{avg}T\Omega }{B}\right)}^{2}}}}sin\left(\Omega t+\xi \right).$$

Equation (9) shows that the *L_Ro_*(*t*) varies sinusoidally with angular velocity Ω and amplitude *A_Ro_*. Thus, by using the frequency, periodicity, and amplitude characteristics of *L_Ro_*(*t*), the rotating speed Ω, the axial clearance *L*, and the tilt angle *α* of the rotor can be estimated by the following equations:

(10) $$\hat{\Omega }=FT\left[L_{Ro}\left(t\right)\right],$$

(11) $$\hat{L}=\frac{\hat{\Omega }}{2\pi m}\int_{t-\pi m/\hat{\Omega }}^{t+\pi m/\hat{\Omega }}L_{Ro}\left(t\right)dt,$$

(12) $$\hat{\alpha }=\arctan\left[\frac{A_{Ro}}{r}{\left(\sqrt{1+{\left(\frac{f_{avg}}{B}T\hat{\Omega }\right)}^{2}}\right)}^{-1}\right],$$

where the symbol ^ denotes the estimated value of the parameter, *FT* represents solving the angular frequency of *L_Ro_*(*t*) by using frequency analysis method such as Fourier Transform, *m* is a positive integer (in this paper we set *m* = 1 for following calculations), and *r* in Equation (12) is a pre-determined value during the probe installation. Note that the *L_Ro_*(*t*) can be obtained by (*dψ*(*t*)/*dt*)·*Tc*/(4*πB*), where *ψ*(*t*) is the phase of the measured FSI signal *s*(*t*) and which can be given by the Hilbert transform [26].

In practice, the perfect parallel installation of the probe is difficult, i.e., *β* is small but nonzero. To analyze the influence of *β* on the multi-parameter measurement, we expand Equation (7) by using the first-order approximation at the point of tan*β* = 0

(13) $$\begin{array}{cc} L_{Ro}^{1}\left(\tan\beta \right) & =\sum_{n=0,1}{\frac{{\left(\tan\beta \right)}^{n}}{n!}\left[\frac{\partial^{n}L_{Ro}}{\partial {\left(\tan\beta \right)}^{n}}\right]|}_{\tan\beta =0}\\ & =L+r\tan\alpha \sqrt{1+{\left(\frac{f_{avg}T\Omega }{B}\right)}^{2}}\sin\left(\Omega t+\xi \right)+K\tan\beta , \end{array}$$

where:

(14) $$K=\tan\alpha \cdot \sin\left(\Omega t-\varphi \right)\left\{\left[L-r\tan\alpha \cdot \cos(\Omega t)\right]\left(1-\frac{f_{avg}T\Omega }{B}\right)+\frac{f_{avg}T\Omega }{B}r\tan\alpha \sin\left(\Omega t\right)\right\}.$$

It is easy to prove that the *K* has a period of 2*π*/Ω and a mean value of 0, which indicates that the angle *β* does not affect the values of $\hat{\Omega }$ and $\hat{L}$. Comparing with Equation (9), the term *K*tan*β* in Equation (13) can be regarded as a perturbation on the amplitude *A_Ro_*, which will lead to an error on $\hat{\alpha }$. We calculate the relative error $e_{\hat{\alpha }}$, which has an upper bound as follow (see Appendix A for details):

(15) $$e_{\hat{\alpha }}\leq \frac{\tan\beta }{r}\left(L+2r\tan\alpha \right).$$

Equation (15) indicates that the relative error is related to the parameters *β*, *r*, *L*, *α*. To achieve a certain level of estimation accuracy, it is better to specify suitable probe installation parameters before measurement according to Equation (15). For instance, $e_{\hat{\alpha }}$ < 5% requires (*L*/*r* + 2tanα) < 1 for *β* = 3°, which means even for a large tilt angle α = 5°, the estimation accuracy can be easily guaranteed by installing the probe at a location where *L*/*r* < 0.8. Note that Equation (15) holds for arbitrary *φ* ∈ (0, 2*π*).

## 3. Experiments and Results

### 3.1. Experimental Setup

**Figure 4:**
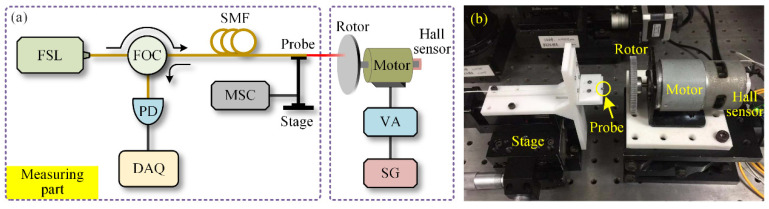
(**a**) Schematic of the experimental system, and (**b**) photograph of the rotational motion setup. (FSL: frequency-swept laser; FOC: fiber-optic circulator; SMF: single-mode fiber; PD: photodetector; DAQ: data acquisition system; MSC: motorized stage controller; SG: signal generator; VA: voltage amplifier.).

To verify the proposed method, an experimental system was built, as shown in Figure 4. The FSL (Arcadia Optronix, GC-760001c, frequency-swept range 196,250 GHz-191,386 GHz, sweeping period 0.486 ms, coherent length < 95 m.) was adopted as the laser source, and the FSI signal detected by the PD was acquired by the DAQ with a sampling rate of 5 MSa/s. The probe was a cleaved single-mode fiber (output laser beam divergence was about 8°). The position of the probe can be adjusted by the motorized stage (adjusting accuracy was better than 1 μm). The rotational motion of the aluminum rotor was provided by the motor. The driving voltage of the motor was generated by the signal generator (DG4102, RIGOL) and amplified by the voltage amplifier (HAS 4011, NF Corporation). The rotational speed was monitored by the Hall sensor installed at the end of the motor.

### 3.2. Results and Analysis

**Figure 5:**
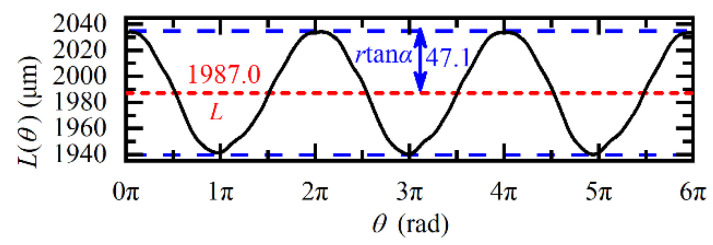
Variation of the actual distance *L*(*θ*) under the condition of Ω = 0 rad/s.

To verify the effectiveness of the proposed method, it was necessary to know the true values of the rotor parameters (i.e., the true values of *L*, *α*, and Ω) in the experimental system. For this purpose, before loading the driving voltage on the motor, we manually rotated the rotor three cycles and measured the actual clearance *L*(*θ*) = *L* + *r*tan*α*cos(*θ*) under the condition of Ω = 0 rad/s by using the fiber probe (*r* = 2 mm, *β* < 2°). The measured *L*(*θ*) is shown in Figure 5, and the true values of *L* and *r*tan*α* can be obtained as *L* = 1987.0 μm and *r*tan*α* = 47.1 μm. As *r* = 2 mm, the true value of the tilt angle *α* can be determined as arctan (*r*tan*α*/*r*) = 1.35°. Thus, *L* = 1987.0 μm, *α* = 1.35°, and as for Ω, it was determined by the Hall sensor when the rotor rotates.

**Figure 6:**
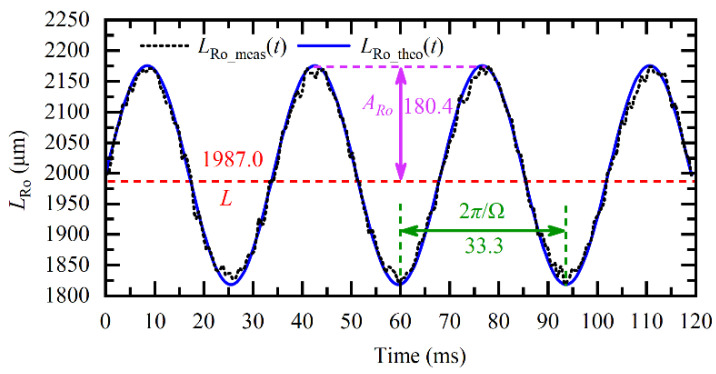
Measured *L*_Ro_meas_(*t*) and theoretical *L*_Ro_theo_(*t*) under the condition of Ω = 60*π* rad/s.

According to Equation (9), after loading the driving voltage, there will be a sinusoidal modulation on the *L*_Ro_(*t*) when the rotor rotates. To verify Equation (9), the angular velocity Ω was set to 60*π* rad/s, and the experimental and theoretical values are plotted in Figure 6. The measured *L*_Ro_meas_(*t*) was obtained from the phase slope *dψ*(*t*)/*dt* of the raw FSI signal, and the theoretical *L*_Ro_theo_(*t*) was calculated according to Equation (9). It was found that the *L*_Ro_meas_(*t*) matches well with the *L*_Ro_theo_(*t*). Moreover, comparing Figure 5 and Figure 6, it was also found, due to the rotation-induced Doppler effect, the amplitude of *L*_Ro_meas_(*t*) increases to 180.4 μm, which was 3.83 times that of *L*(*θ*), and this value agrees well with the theoretical amplitude modulation coefficient (1 + (*f_avg_T*Ω/*B*)^2^)^1/2^ = 3.79 given by Equation (9).

According to the dynamic FSI model of rotors in Equation (9), the direct component (DC component), frequency, and amplitude of the *L*_Ro_(*t*) are related to the axial distance *L*, angular velocity Ω, and tilt angle *α*. Therefore, when the *L*_Ro_meas_(*t*) was obtained, the multiple parameters could be calculated according to Equations (10)–(12). For example, using the *L*_Ro_meas_(*t*) in Figure 6, the *L* and Ω were respectively estimated to be 1987.0 μm and 60*π* rad/s. Besides, using *A*_Ro_ = 180.4 μm, the α was estimated to be 1.36°, according to Equation (12), which was consistent with the true value of 1.35°. The relative measurement error of α was 1%, less than the theoretical upper bound $e_{\hat{\alpha }}=\tan\beta \left(L/r+2r\tan\alpha \right)=3.7\%$ given by Equation (15).

**Figure 7:**
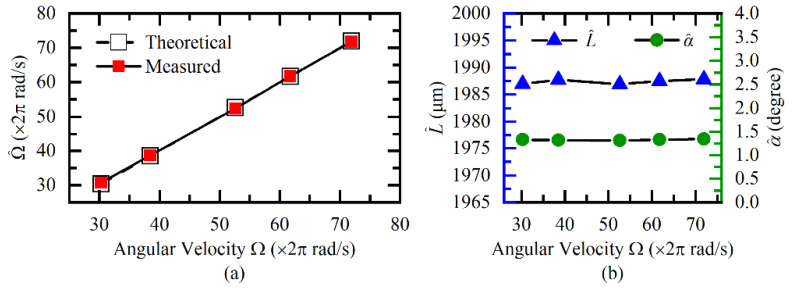
Measurement results of (**a**) angular velocity, (**b**) axial clearance, and tilt angle under different angular velocities.

To further verify the adaptability of the proposed method at different Ω, *L*, and *r*, a series of experiments were carried out. First, we kept the position of the fiber probe unchanged and set Ω from 60*π* rad/s to 160*π* rad/s. The multi-parameter measurement results are shown in Figure 7. In Figure 7a, the theoretical Ω was given by the Hall sensor, and the estimated $\hat{\Omega }$ is calculated by the proposed method. It can be seen that the angular velocity was accurately recovered, with a relative error of about 1.5%. As expected, the estimated $\hat{L}$ and $\hat{\alpha }$ in Figure 7b remained constant, and their relative errors were less than 0.04% and 2.53%, respectively.

**Figure 8:**
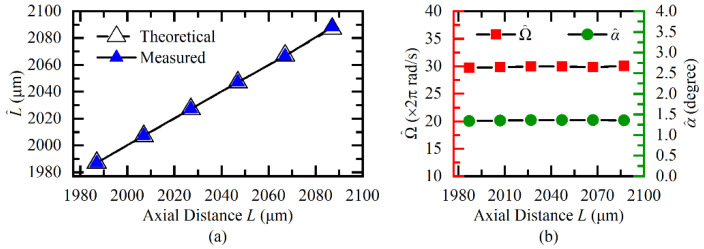
Measurement results of (**a**) axial clearance, (**b**) angular velocity, and tilt angle under different axial clearances.

Then, we reset the Ω to the fixed value of 60*π* rad/s and changed the *L* by using the motorized stage. The *L* was changed from 1987 μm to 2087 μm with a step of 20 μm. The measurement results are shown in Figure 8. In Figure 8a, the theoretical *L* is the sum of the displacement and the initial clearance 1987 μm, and the $\hat{L}$ is obtained according to Equation (11). It was evident that the estimated $\hat{L}$ was consistent with the theoretical *L*, and the maximum relative measurement error was less than 0.06%. It can be seen from Figure 8b that the estimated $\hat{\Omega }$ and $\hat{\alpha }$ were independent of the variation of $\hat{L}$. The relative measurement errors of $\hat{\Omega }$ and $\hat{\alpha }$ were respectively less than 0.91% and 1.02%.

**Figure 9:**
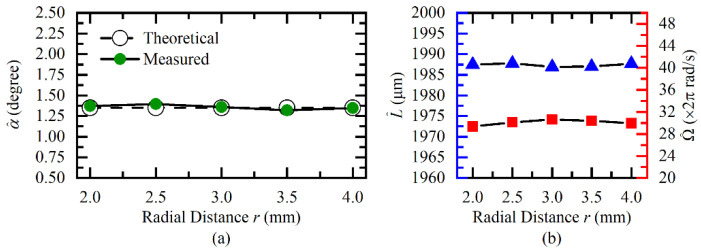
Measurement results of (**a**) tilt angle, (**b**) axial clearance, and angular velocity under different radii.

Next, we fixed the *L* and Ω respectively to 1987 μm and 60*π* rad/s, and changed the fiber probe position *r* from 2 mm to 4 mm with a step of 0.5 mm. Corresponding measurement results under different positions are illustrated in Figure 9. The maximum estimation relative errors of $\hat{\alpha }$, $\hat{L}$ and $\hat{\Omega }$, shown in Figure 9 were less than 3.42%, 0.04%, and 2.11%, respectively. The proposed method still performed well in this case.

## 4. Conclusions

In this paper, we presented a method for simultaneously measuring the axial clearance, rotational speed, and tilt angle of the rotor by using the Doppler effect of FSI. The measurement steps are as follows: first, obtained the instantaneous phase *ψ*(*t*) of FSI signal by using the Hilbert transform and calculate the dynamic distance *L_Ro_*(*t*) = (*dψ*(*t*)/*dt*)·*Tc*/(4*πB*) according to Equation (3); then, estimated the angular velocity Ω, axial clearance *L*, and tilt angle *α* by using the frequency, direct component and amplitude of *L_Ro_*(*t*) according to Equations (10)–(12). Considering the practical non-parallel installation of the probe, we analyzed the influence of the non-parallel installation on the measurement error and found that the measurement error could be eliminated by installing the probe at a proper location (*L*/*r* < 0.8). The multi-parameter measurement experiments were carried out to verify the proposed method, and the relative errors of the axial clearance, rotational speed, and tilt angle were respectively lower than 0.1%, 2.2%, and 3.5%. The main difference between the previously reported FSI-based works and this work is that we used the Doppler error as a tool rather than an obstacle. This idea reduced the bulkiness of conventional, integrated multiple parameter measurement systems. Besides, the optic-fiber probe of our system is small in size, light-weight, and bendable, which is very suitable for the space-confined rotatory machines, such as generators, motors, and gear-sets. Moreover, combining our method with the reported fiber-optic radial clearance measurement method would enable a more comprehensive multi-parameter measurement of rotors.

## Appendix A. Upper Bound of the Relative Error in the Estimation of *α*

According to Equation (7), we can consider *L_Ro_* as a function of tan*β*:

(A1) $$L_{Ro}\left(\tan\beta \right)=\frac{L+r\tan\alpha \sqrt{1+{\left(\frac{f_{avg}T\Omega }{B}\right)}^{2}}sin\left(\Omega t+\xi \right)-\frac{f_{avg}T\Omega }{B}\left(L-z_{A}\right)\tan\alpha \tan\beta \sin(\Omega t-\varphi )}{\left[1-\tan\alpha \tan\beta \sin(\Omega t-\varphi )\right]/\sqrt{1+\tan^{2}\beta }},$$

where:

(A2) $$z_{A}=\frac{r\sin\alpha \cos(\Omega t)-L\sin\alpha \tan\beta \cos(\Omega t-\varphi )}{\cos\alpha -\sin\alpha \tan\beta \cos(\Omega t-\varphi )}=\frac{r\tan\alpha \cos(\Omega t)-L\tan\alpha \tan\beta \cos(\Omega t-\varphi )}{1-\tan\alpha \tan\beta \cos(\Omega t-\varphi )}.$$

For small angle *β*, *L_Ro_*(tan*β*) can be approximated by the first-order Taylor series expansion at tan*β =* 0

(A3) $$\begin{array}{cc} L_{Ro}^{1}\left(\tan\beta \right) & ={\sum_{n=0,1}\frac{{\left(\tan\beta \right)}^{n}}{n!}\left[\frac{\partial^{n}L_{Ro}}{\partial {\left(\tan\beta \right)}^{n}}\right]|}_{\tan\beta =0}\\ & =L+r\tan\alpha \sqrt{1+{\left(\frac{f_{avg}T\Omega }{B}\right)}^{2}}\sin\left(\Omega t+\xi \right)+{\frac{\partial L_{R_{0}}}{\partial \tan\beta }|}_{\tan\beta =0}\tan\beta , \end{array}$$

we can obtain:

(A4) $$\begin{array}{cc} {\frac{\partial L_{R_{0}}}{\partial \tan\beta }|}_{\tan\beta =0} & =\tan\alpha \sin\left(\Omega t-\varphi \right)\\ & \left[L\left(1-\frac{f_{avg}T\Omega }{B}\right)+r\tan\alpha \sqrt{1+{\left(\frac{f_{avg}T\Omega }{B}\right)}^{2}}\sin\left(\Omega t+\xi \right)+\frac{f_{avg}T\Omega }{B}r\tan\alpha \cos(\Omega t)\right]\\ & =\tan\alpha \sin\left(\Omega t-\varphi \right)\\ & \left[L\left(1-\frac{f_{avg}T\Omega }{B}\right)-r\tan\alpha \cos(\Omega t)+\frac{f_{avg}T\Omega }{B}r\tan\alpha \sin\left(\Omega t\right)+\frac{f_{avg}T\Omega }{B}r\tan\alpha \cos(\Omega t)\right]\\ & =\tan\alpha \sin\left(\Omega t-\varphi \right)\left\{\left[L-r\tan\alpha \cos(\Omega t)\right]\left(1-\frac{f_{avg}T\Omega }{B}\right)+\frac{f_{avg}T\Omega }{B}r\tan\alpha \sin\left(\Omega t\right)\right\}. \end{array}$$

Thus, we have

(A5) $$\begin{array}{cc} L_{Ro}^{1}\left(\tan\beta \right)= & \underset{L_{Ro}}{\underset{︸}{L+r\tan\alpha \sqrt{1+{\left(\frac{f_{avg}T\Omega }{B}\right)}^{2}}\sin\left(\Omega t+\xi \right)}}\\ & +\underset{K}{\underset{︸}{\tan\alpha \sin\left(\Omega t-\varphi \right)\left\{\left[L-r\tan\alpha \cos(\Omega t)\right]\left(1-\frac{f_{avg}T\Omega }{B}\right)+\frac{f_{avg}T\Omega }{B}r\tan\alpha \sin\left(\Omega t\right)\right\}}}\tan\beta , \end{array}$$

where it can be found that the first term is the distance *L_Ro_* under the condition of *β* = 0. Thus, the term *K*tan*β* induced by a small *β* can be regarded as a time-varying perturbation on *L_Ro_*, which would lead to a change in the amplitude *A_Ro_* of *L_Ro_*. Thus, the change of *A_Ro_* is within the range from −|*K*tan*β*|_max_ to |*K*tan*β*|_max_. Here, we define *e* as

(A6) $$e=\frac{\tan{\hat{\alpha }}^{\prime }}{\tan\hat{\alpha }}\leq \frac{\frac{A_{Ro}+{\left|K\tan\beta \right|}_{\max}}{r}{\left(\sqrt{1+{\left(\frac{f_{avg}}{B}T\hat{\Omega }\right)}^{2}}\right)}^{-1}}{\frac{A_{Ro}}{r}{\left(\sqrt{1+{\left(\frac{f_{avg}}{B}T\hat{\Omega }\right)}^{2}}\right)}^{-1}}=1+\frac{{\left|K\tan\beta \right|}_{\max}}{A_{Ro}},$$

where $\hat{\alpha }$ and ${\hat{\alpha }}^{\prime }$ are respectively the estimation of α under the conditions of β=0 and β≠0.

Using |*a* + *b*| ≤ |*a*| + |*b*|, |*ab*| = |*a*||*b*|, |sin(Ωt)|≤1 and |cos(Ωt)|≤1, we can obtain:

(A7) $$\begin{array}{cc} \frac{{\left|K\tan\beta \right|}_{\max}}{A_{Ro}} & \leq \frac{\left|\tan\alpha \sin\left(\Omega t-\varphi \right)\left\{\left[L-r\tan\alpha \cos(\Omega t)\right]\left(1-\frac{f_{avg}T\Omega }{B}\right)+\frac{f_{avg}T\Omega }{B}r\tan\alpha \sin\left(\Omega t\right)\right\}\tan\beta \right|}{r\tan\alpha \sqrt{1+{\left(\frac{f_{avg}T\Omega }{B}\right)}^{2}}}\\ & \leq \frac{\tan\beta }{r}\left\{\left|\left[L-r\tan\alpha \cos(\Omega t)\right]\left[1-\frac{f_{avg}T\Omega }{B}\right]\right|+\left|\frac{f_{avg}T\Omega }{B}r\tan\alpha \right|\left|\sin\left(\Omega t\right)\right|\right\}\frac{\left|\sin\left(\Omega t-\varphi \right)\right|}{\sqrt{1+{\left(\frac{f_{avg}T\Omega }{B}\right)}^{2}}}\\ & \leq \frac{\tan\beta }{r}\left\{\left|\left[L-r\tan\alpha \cos(\Omega t)\right]\right|\cdot \frac{\left|1-\frac{f_{avg}T\Omega }{B}\right|}{\sqrt{1+{\left(\frac{f_{avg}T\Omega }{B}\right)}^{2}}}+\frac{\frac{f_{avg}T\Omega }{B}}{\sqrt{1+{\left(\frac{f_{avg}T\Omega }{B}\right)}^{2}}}r\tan\alpha \right\}\\ & \leq \frac{\tan\beta }{r}\left\{\left|L-r\tan\alpha \cos(\Omega t)\right|+r\tan\alpha \right\}\\ & \leq \frac{\tan\beta }{r}\left\{\left|L\right|+\left|-r\tan\alpha \right|\cdot \left|\cos(\Omega t)\right|+r\tan\alpha \right\}\\ & \leq \frac{\tan\beta }{r}\left(L+2r\tan\alpha \right). \end{array}$$

It can be seen from Equation (A7), the sin(Ω*t*−*φ*) term that contains the deflection angle *φ* is amplified and taken as 1, thus Equation (A7) holds for arbitrary *φ* ∈ [0, 2*π*). Therefore, whatever the *φ* is, the relative estimation error of $\hat{\alpha }$ caused by a small *β* satisfies:

(A8) $$e_{\hat{\alpha }}=\frac{{\hat{\alpha }}^{\prime }-\hat{\alpha }}{\hat{\alpha }}=\frac{\arctan\left(e\tan\hat{\alpha }\right)-\hat{\alpha }}{\hat{\alpha }}\leq \frac{\arctan\left\{\underset{\geq 1}{\underset{︸}{\left[1+\frac{\tan\beta }{r}\left(L+2r\tan\alpha \right)\right]}}\underset{\geq 0}{\underset{︸}{\tan\hat{\alpha }}}\right\}-\hat{\alpha }}{\hat{\alpha }}$$

Mathematically, for the following function *y*(*p*, *q*),

(A9) y(p,q)=pq−arctan(ptanq),

we have

(A10) $$\begin{array}{cc} \frac{\partial y}{\partial q} & =p-\frac{p\sec^{2}(q)}{p^{2}\tan^{2}(q)+1}\\ & =\frac{p^{3}\tan^{2}(q)-p\tan^{2}(q)+p+\underset{-p}{\underset{︸}{\left[p\tan^{2}(q)-p\sec^{2}(q)\right]}}}{p^{2}\tan^{2}(q)+1}\\ & =\frac{p\left(p^{2}-1\right)\tan^{2}(q)}{p^{2}\tan^{2}(q)+1}, \end{array}$$

obviously, when *p* ≥ 1 and *q* ≥ 0, we have ∂*y*/∂*q* ≥ 0. So, we have

(A11) y(p,q)=pq−arctan(ptanq)≥y(p,0)=0.

Therefore, in Equation (A8), if we define

(A12) $$\left\{ \begin{array}{l} p=\left[1+\frac{\tan\beta }{r}\left(L+2r\tan\alpha \right)\right],\\ q=\hat{\alpha }, \end{array}\right.$$

according to Equation (A11), Equation (A8) can be simplified as:

(A13) $$\begin{array}{cc} e_{\hat{\alpha }} & \leq \frac{\arctan\left\{\left[1+\frac{\tan\beta }{r}\left(L+2r\tan\alpha \right)\right]\tan\hat{\alpha }\right\}-\hat{\alpha }}{\hat{\alpha }}\\ & \leq \frac{\hat{\alpha }\left[1+\frac{\tan\beta }{r}\left(L+2r\tan\alpha \right)\right]-\hat{\alpha }}{\hat{\alpha }}\\ & =\frac{\tan\beta }{r}\left(L+2r\tan\alpha \right). \end{array}$$

It needs to point out that, from Equation (A6) to Equation (A13), we consider the case of *A_Ro_* increased by |*K*tan*β*|_max_. For the case of *A_Ro_* decreased by −|*K*tan*β*|_max_, it is easy to get $e_{\hat{\alpha }}\geq -\tan\beta \left(L+2r\tan\alpha \right)/r$. So the $e_{\hat{\alpha }}$ in Equation (A13) can be regarded as the absolute value of the relative error.
